# Pioglitazone and Endothelial Dysfunction: Pleiotropic Effects and Possible Therapeutic Implications

**DOI:** 10.3797/scipharm.1407-16

**Published:** 2014-08-18

**Authors:** Miroslav Radenković

**Affiliations:** Department of Pharmacology, Clinical Pharmacology and Toxicology; Faculty of Medicine; University of Belgrade; PO Box 38; 11129 Belgrade; Serbia.

**Keywords:** Pioglitazone, Endothelial dysfunction, Diabetes, Nitric oxide, PPARy

## Abstract

The vascular endothelium has a central role in the modulation of vascular tone with associated antioxidant, anti-inflammatory, pro-fibrinolytic, anti-adhesive, and anticoagulant effects. This is primarily accomplished by the timely release of endothelial autacoids. On the other hand, endothelial dysfunction (ED) provoked by insulin resistance has been linked with reduced nitric oxide bioavailability, increased production of reactive oxygen species, and alterations of endothelial regeneration. Pioglitazone is classified as an insulin-sensitizing, anti-hyperglycemic agent. The mechanism of action associated with pioglitazone includes the activation of peroxisome proliferator-activated receptor-gamma with stable improvement in glycemic control in diabetic patients. Today, it is known that apart from the beneficial effects on glucose homeostasis, pioglitazone exerts several pleiotropic effects, including the improvement of ED. Thus, the aim of this article was to summarize the current knowledge related to signaling mechanisms of the pioglitazone-induced improvement or reversal of ED. The relevant clinical studies and possible therapeutic implications connected to pioglitazone-related action on the endothelium were analyzed too.

## Introduction

Thiazolidinediones, as an insulin-sensitizing group of drugs, have been shown to exert beneficial effects on the cardiovascular system independently of their action on glucose and insulin sensitivity [1]. They have been demonstrated to be effective alone or in combination with a sulfonylurea, metformin, or insulin [2]. The peroxisome proliferator-activated receptor-gamma (PPARy) agonist, pioglitazone, belongs to the insulin-sensitizing group of drugs, which is used in the treatment of type 2 diabetes mellitus (T2DM) [3]. Insulin resistance and hyperglycemia contribute to the development and progression of atherosclerosis within a complex milieu of interrelated risk factors, which include hypertension, dyslipidemia, chronic subclinical inflammation, ED, and abnormalities in coagulation and fibrinolysis [4]. Insulin resistance is typically present for some years before diagnosis, manifested as diminished stimulation of glucose transport in muscle and adipose tissue and inadequate suppression of glucose production in the liver in response to insulin [5]. Although PPARy activation plays an important role in glucose metabolism by enhancing insulin sensitization, the activation of quoted ligand-activated transcription factor inhibits adhesion cascades and detrimental vascular inflammatory events with a distinctive role in regulating the physiology and expression of endothelial NO-synthase (eNOS), thus resulting in enhanced generation of vascular (nitric oxide) NO [6]. Knowing that NO is the most important endothelium-derived relaxing autacoid and that there is a positive correlation among insulin and the up-regulation of eNOS [7], the additional mechanisms related to the activation of PPARy may be significant for cardiovascular disorders linked with diabetes mellitus, too. Accordingly, even though pioglitazone is known to exert renoprotective effects in diabetic nephropathy at doses that normalize glycemia, it has also been reported that at low doses that do not normalize glycemia, pioglitazone administration in Zucker diabetic fatty rats was associated with the normalization of the renal levels of connective tissue growth factor and fibronectin, tumor necrosis factor-a (TNFa), interleukin-6 (IL-6), and monocyte chemotactic protein-1, megalin, the proliferating cell nuclear antigen/caspase-3 ratio, vascular endothelial growth factor (VEGF), and the ratio between endothelial and inducible NOS [8]. The beneficial effects of short-term, low-dosage pioglitazone on ED in regards to increasing adiponectin expression and decreasing low-grade inflammation in T2DM patients were reported as well [9]. These and other similar findings have led to the hypothesis that pioglitazone could exert vasculoprotective effects that are independent of its metabolic action involving the activation of PPARy [2].

The endothelium is composed of a monolayer of cells that line the lumen of blood vessels and form a physical barrier between circulating blood and the vascular smooth muscle cells [10]. The important role of the endothelium in regulating vascular tone, inflammatory responses, coagulation, and thrombocyte adhesion is now recognized [3]. This is for the most part achieved by the balanced release of endothelial relaxing factors, such as NO, prostacyclin, or endothelium-derived hyperpolarizing factor [11–13]. Oppositely, ED, as a pathological process, is characterized by the impaired regulation of vascular tone as a result of reduced eNOS activity, lack of cofactors for NO synthesis, attenuated NO release, or increased NO degradation [14]. ED and abnormal angiogenesis are considered as central orchestrators of vascular complications in diabetes [15]. It is well-established that atherosclerosis is a complex process that progresses through various functional and morphological alterations in the vessel wall, cuminating in overt cardiovascular disease [16]. Macroangiopathy, as a pathological condition, is known to be directly correlated with atherosclerosis, which is in turn associated with vascular ED and reduced NO. Diabetic macroangiopathy, with the consequential peripheral vascular disease, stroke, or coronary heart disease, can be marked as the leading cause of mortality and disability due to T2DM [17]. This is supported by the fact that an accelerated progression of the atherosclerotic process can be detected in patients with T2DM [18]. Oppositely to microangiopathies, where the strong relation to hyperglycemia is confirmed, the link between hyperglycemia and macroangiopathy is uncertain, at least in terms of the possibility of reducing macrovascular morbidity merely by reducing hyperglycemia [10].

Taking into account previous considerations, the aim of this article was to summarize up-to-date knowledge related to the signaling mechanisms of pioglitazone-induced improvement or reversal of ED. Thus, the evidence acquisition included MEDLINE database screening, and the relevant experimental and clinical studies from the last five years in regards to the possible therapeutic implications connected to pioglitazone-related action on endothelial cells were analyzed.

## Pioglitazone-Related Findings Obtained on Animals

Omae et al. (2013) [19] tested the hypotheses that homocysteine (Hcy) impairs endothelium-dependent vasodilation of the retinal arterioles by increasing oxidative stress, and that co-treatment with pioglitazone prevents the resulting ED via activation of PPARy. An elevated level of total Hcy is an important risk factor for premature atherosclerosis and resulting cardiovascular, cerebrovascular, and peripheral arterial disease [20, 21]. In accordance, elevated Hcy levels were shown to be associated with retinal vascular diseases including diabetic retinopathy [22]. So far, investigations on the effects of Hcy in endothelial cells have emphasized the presence of cellular stress and cellular damage, thus indicating the progression of ED, mostly via production of reactive oxygen species [23, 24]. Hence, by using an isolated vessel preparation, Omae et al. (2013) [19] examined the endothelium-dependent and NO-mediated dilation of pig retinal arterioles with and without Hcy, as well as the possibility that key vascular signaling molecules characterizing oxidative stress are involved in the Hcy-mediated effects. To evaluate the capacity of pioglitazone to reduce the effect of Hcy on NO-mediated vasodilation, vessels were treated with Hcy combined with pioglitazone (0.5 μM). As a result, bradykinin and A23187 dilated the retinal arterioles in a dose-dependent manner, thus indicating the endothelium-and NO-dependent mechanism. Hcy (1 mM) significantly reduced both the bradykinin and A23187 relaxant effects (P < 0.001 and P = 0.017, respectively). TEMPOL (cell-permeable superoxide scavenger, 1 mM) prevented the detrimental effect of Hcy on bradykin-induced vasodilation, and this finding was also detected in vessels treated with NAD(P)H oxidase inhibitor apocynin (100 μM). Likewise, the coadministration of Hcy and pioglitazone prevented the negative action of Hcy on the bradykinin-evoked relaxant effect. This was additionally confirmed when the inhibitory effect of Hcy on the vasodilatory response to bradykinin did not change in the presence of the PPARy inhibitor GW9662 (10 μM). It was concluded that pioglitazone protects against Hcy-induced ED in retinal arterioles, possibly through the activation of PPARy and the reduction of oxidative stress.

A stroke is one of the principal causes of a death outcome and transient or permanent disability. However, the role of PPARy and its co-regulators in cerebrovascular ED related to stroke is still under investigation. In the recent experimental investigation on different types of knockout mice conducted by Yin et al. (2013) [25], it has been shown that the activation of PPARy by pioglitazone significantly inhibited both oxygen–glucose deprivation-induced cerebral vascular endothelial cell death and middle cerebral artery occlusion-triggered cerebrovascular damage. In this investigation, focal cerebral ischemia was induced in mice by transient intraluminal middle cerebral artery occlusion for 30 min followed by 24 h reperfusion. In accordance, to mimic the ischemia-like conditions *in vitro*, mouse cerebral vascular endothelial cell cultures were exposed to oxygen–glucose deprivation. Pioglitazone was shown to exert a dose-dependent positive action on endothelial cell death inhibition. A potentiation of pioglitazone-mediated cytoprotection after 16 h of oxygen–glucose deprivation exposure was detected as well. In addition, pioglitazone-mediated cytoprotection after oxygen–glucose deprivation was confirmed to be a PPARy-dependent process. Consistent with previous findings, the authors have demonstrated that selective PPARy genetic deletion in vascular endothelial cells resulted in increased cerebrovascular permeability and brain infarction in mice after focal ischemia. On the other hand, pioglitazone significantly attenuated ischemia-triggered increases in cerebrovascular permeability. The obtained data demonstrated that the loss of PPARy function in the cerebral vasculature exacerbates ischemic cerebrovascular and brain damage, whereas PPARy activation by pioglitazone most probably plays a vascular protective role in ischemic stroke.

Microvascular alterations are involved in diabetic organ damage including dysregulation, enhanced permeability, and pathologic angiogenesis, as well as capillary rarefaction, which is characterized by functional insufficiency of capillary recruitment and a structural reduction of capillary density [26]. The development of capillary rarefaction may involve various detrimental factors, but the essential role in diabetes can be linked to ED and reduced NO bioavailability, in part via the scavenging actions of oxidative free radicals [27]. Since the reduction of capillary network density occurs early in the development of metabolic syndrome and may be relevant for the precipitation of diabetes, Ashoff et al., (2012) [26] hypothesized that treatment with pioglitazone might prevent diabetic capillary rarefaction in a manner independent of its antihyperglycemic activity. In the conducted investigation, a total of 50 rats with streptozotocin-induced diabetes were treated with pioglitazone for up to 12 weeks. Capillary density was determined both in the heart and skeletal muscle. Thus, a progressive loss of capillaries occurred in the quadriceps muscle regarding the area density, as well as the number of capillaries per myocyte. On the other hand, when treatment had begun 8 days after the induction of diabetes, the development of capillary rarefaction in the skeletal as well as heart muscle was consistently attenuated by pioglitazone. Therefore, pioglitazone has been shown to protect the microvasculature of the skeletal and cardiac muscle from structural decline, although this effect was additionally shown to not be related to the angiogenic actions of vascular endothelial growth factor or glucose control.

Since vascular research in animal models of metabolic syndrome is relatively limited, Mendizábal et al. (2011) [28] aimed to investigate the effects of rosiglitazone and pioglitazone on the reactivity of the isolated aorta and mesenteric resistance arteries from the obese spontaneously hypertensive rats (SHR). Thus, the reactivity of the mesenteric resistance arteries and the aorta from the SHR and control rats to cumulative concentrations of phenylephrine, acetylcholine, and sodium nitroprusside (SNP) was myographically analyzed. As expected, phenylephrine, acetylcholine, and SNP dose-response curves were impaired to different extents in the arteries of the obese SHR.

Incubation with pioglitazone or rosiglitazone, or oral treatment with rosiglitazone, improved acetylcholine and SNP curves to different extents in all vessels. In regards to pioglitazone only, the presence of this agent in the organ bath significantly increased the maximal relaxing ability of acetylcholine in the mesenteric resistance arteries of obese rats, which occurred at an acetylcholine concentration of 0.3 μM. Moreover, both kinds of obese SHR’ arteries relaxed significantly more to SNP when the used glitazones were present, but the improvement was more latent in mesenteric resistance arteries. Thus, the analyzed results suggest the beneficial effects both on endothelium-dependent, as well endothelium-independent, relaxing mechanisms on the investigated blood vessels. In other words, it can be proposed that the applied glitazones positively affected both endothelial and smooth muscle vascular cells in the used experimental model of metabolic syndrome.

## Pioglitazone-Related Findings Obtained on Humans

In the recent randomized, double-blind, and prospective study of Yu et al. (2013) [17], it has been determined that pioglitazone ameliorated ED in subjects with impaired glucose regulation among first-degree relatives of T2DM patients. The test group consisted of 107 first-degree relatives (30–59 years of age) that were diagnosed with impaired glucose regulation or impaired fasting glucose for the first time or before, but without treatment or already off of treatment for more than three months. Impaired fasting glucose subjects whose blood glucose was still above the level after one month of exercise were randomized to receive pioglitazone (15 mg/day) or a vehicle for 12 weeks. It is known that in the clinical setting, endothelial function may be measured indirectly by using the technique of brachial artery flow-mediated dilation (FMD) [1]. Hence, endothelial function was assessed as endothelium-dependent and endothelium-independent vasodilation by means of ultrasonographic measuring of the brachial artery’s inner diameter changes. The measurements were done at baseline, before, and after reactive hyperemia, or sublingual nitroglycerin. Hence, after the pioglitazone treatment, endothelium-dependent vasodilation and blood NO were significantly improved in the treatment group. In particular, in the pioglitazone-treated participants, NO concentration was notably increased from 38.7 ± 11.4 μg/l to 53.3 ± 19.3 μg/l, while in the control group, this trend was not evident (before: 41.2 ± 16.7 μg/l; after: 39.6 ± 12.5 μg/l). Furthermore, the changes in endothelium-dependent vasodilation were negatively correlated with the changes in the calculated area under the curve of insulin (r = -0.66), but positively with NO (r = 0.73) and the homeostasis model assessment of beta cell function (r = 0.72). Moreover, both of these indicators were shown to be independent parameters for the improvement of ED. The results of this study suggest that pioglitazone may play a role in the prevention of atherosclerosis and T2DM in impaired glucose regulation patients, most probably by increasing the production of NO in endothelial cells.

It is known that cardiovascular homeostasis is considerably modulated by endothelial progenitor cells (EPC) that are potent biologic markers of endothelial function. EPC are circulating cells with the ability to differentiate into mature endothelium with notable participation in the neoangiogenesis reparative processes after endothelial damage. Therefore, the aim of research reported by Spigoni et al. (2012) [29] was to evaluate whether the addition of pioglitazone *in vitro* can substantiate benefits in EPC obtained from impaired glucose tolerance subjects in terms of apoptosis, viability, and tube formation capacity. The authors also sought to investigate *in vitro* potential changes in EPC proinflammatory molecule expression in the presence of pioglitazone. Hence, early and late-outgrowth EPC were obtained from the peripheral blood mononuclear cells of 14 impaired glucose tolerance subjects. Inclusion criteria were impaired glucose tolerance males (n=7) or females (n=7) aged 18–65 years (mean age = 58 ± 6 years) with adequately defined exclusion criteria. The *in vitro* action of pioglitazone (10 μM), with or without the antagonist of PPARy (GW9662, 1 μM), was assessed on EPC’ viability, apoptosis, ability to form tubular-like structures, and pro-inflammatory molecule expression. Thus, pioglitazone improved early and late-outgrowth EPC viability by 65% and 161%, respectively, compared to the vehicle. The obtained action was mediated via PPARy since the effect was blocked by GW9662. Furthermore, the addition of pioglitazone improved the capacity of EPC to form tubular-like structures expressed as the number of closed circles formed by early and late-outgrowth EPC, as well as total tube length, tube number, and number of sprouts in early and late-outgrowth EPC compared to the control. Once more, the observed effects were dependent upon PPARy, which was also the case with the next findings that pioglitazone reduced the TNFa gene and protein expression, as well as the expression of intercellular adhesion molecule-1 (ICAM-1) and vascular cell adhesion molecule-1 (VCAM-1) in both early and late-outgrowth EPC compared to the control. The authors have concluded that the conducted preclinical study showed that pioglitazone improved the *in vitro* angiogenic capacity of EPC isolated from impaired glucose tolerance subjects and reduced inflammation as well, thus supporting the potential implication of pioglitazone as a cardiovascular protective agent.

In accordance with the previous study, Kampoli et al. (2012) [30] assessed the effects of pioglitazone and perindopril (ACE inhibitor) individually on circulating EPC, as well as on the inflammatory process and oxidative stress as potential mechanisms associated with the mobilization of EPC. In the reported parallel study, 50 patients with T2DM were recruited randomly to receive either pioglitazone (15 mg/day) or perindopril (4 mg/day) in addition to their conventional therapy for one month. Endothelial function was assessed by FMD by ultrasound in the brachial artery, while the number of EPC was expressed as the number of cells per total number of cytometric events. In consideration merely of the pioglitazone-induced effects, the number of EPC was not affected (p=0.09), although it showed a trend to increase. The other results indicating ED improvement were positive. Namely, FMD was improved (p=0.04) and the plasma concentration of VEGF was increased (p=0.01), while on the other hand, C-reactive protein and asymmetric dimethylarginine levels were reduced (p=0.04 and p=0.01, respectively). Although it was found that pioglitazone treatment did not significantly affect the number of circulating endothelial cells, it was suggested that this was probably a result of the small study population. The authors proposed that their results support the beneficial role of pioglitazone in terms of inflammation and oxidative stress.

Abnormal proliferation and apoptosis of endothelial cells are involved in ED, damage, and repair [10]. Taking into account that the possible interference of anti-diabetic and cardioprotective agents with endothelial cell proliferation and apoptosis is still under investigation, the study reported by Eriksson et al. (2012) [10] was aimed to investigate the protective mechanisms of several anti-diabetic drugs, including pioglitazone on human coronary artery endothelial cells. Thus, the effect of anti-diabetic and cardioprotective agents on human coronary artery endothelial cell viability, proliferation, and apoptosis was studied. Viability was assessed using Trypan blue and proliferation in 5 mM and 11 mM of glucose was analyzed. Lipoapoptosis of the cells was investigated by determining caspase-3 activity and the subsequent DNA fragmentation after incubation with the free fatty acid palmitate, mimicking diabetic lipotoxicity. As a result, the viability of human coronary artery endothelial cells was significantly augmented after the 48 h-long incubation with pioglitazone (2.5 μM) when compared to the control cells. In addition, pioglitazone significantly decreased the palmitate-induced caspase-3 activation, suggesting a protective effect of the drug against lipotoxicity in human coronary artery endothelial cells. To additionally substantiate the obtained anti-apoptotic effect, the authors investigated an influence of pioglitazone on palmitate-induced DNA fragmentation by analyzing cyto-plasmic DNA-histone nucleosome complexes generated during apoptosis. Co-incubation with pioglitazone significantly countered the palmitate-induced apoptosis, thus confirming the protective effect against lipoapoptosis in human coronary artery endothelial cells. It can be proposed that pioglitazone exerted a direct and beneficial effect on endothelial cell viability and apoptosis, which in turn can additionally increase its clinical value in T2DM patients in whom ED is present.

Since the comparative effects of pioglitazone versus metformin (both insulin sensitizers) in regards to the possible improvement of the cardiovascular prognosis in patients with T2DM are still elusive, the aim of the proposed study by Naka et al. (2012) [18] was to assess and compare the effects of pioglitazone and metformin on vascular endothelial function in patients with uncontrolled T2DM treated only with glimepiride, a sulfonylurea drug. This was an open-labeled and randomized study in which clinical assessment and further evaluation of endothelial function were performed in all patients at baseline and after six months of concomitant treatment with glimepiride + metformin (850 mg b.i.d, n = 17) or glimepiride + pioglitazone (30 mg o.d., n = 17). Eligible participants were between 40 and 80 years old with an HbA_1C_ above 7.0% during monotherapy with glimeperide for at least six months. The exclusion criteria were properly defined and adequately applied too. Endothelial function was assessed in all patients by ultrasonographic assessment of FMD; in other words, by the measurement of endothelium-dependent, shear-induced vasodilation in the right brachial artery in response to hand hyperemia. Sublingual glyceryl trinitrate was administered for the measurement of endothelium-independent vaso-dilatation in the brachial artery. The reported results of this study suggested that in the pioglitazone group of patients, endothelial function, glycemic control, and insulin resistance were significantly improved, while the addition of metformin to glimepiride was associated with a significant decrease in HbA_1C_ only. An improvement in insulin resistance or endothelial function was present, but not significant in the metformin group of patients. Moreover, pioglitazone-induced improvement in FMD did not appear to differ greatly compared with metformin. Finally, an improvement in FMD was not related to glycemic control or improved insulin sensitivity in neither of the investigated groups.

FMD, as a surrogate marker of endothelial function, was also evaluated in another randomized, double-blind, and placebo-controlled trial, which was conducted with the aim to assess the effect of pioglitazone on the vasculature of patients with impaired glucose tolerance [1]. In this investigation, 40 impaired glucose tolerance patients diagnosed by the oral glucose tolerance test (blood glucose 7.8–11.1 mmol/l 2 h after a 75 g load of oral glucose) with no cardiovascular disease were compared with 24 healthy age- and sex-matched controls. Aside from the FMD procedure, adiponectin levels were measured and insulin sensitivity was calculated through the homeostasis model assessment of insulin resistance. Adiponectin is known to be linked to the improvement of glucose metabolism, as well as having beneficial effects on the endothelium, and it is also reduced in T2DM. The impaired glucose tolerance subjects were randomized into the pioglitazone (30 mg) or matched placebo group, and the measurements were performed at baseline and after 12 weeks. Impaired glucose tolerance participants had significantly impaired FMD, reduced diastolic shear stress (confirming microcirculatory dysfunction), and lower high molecular weight adiponectin when compared with the controls. After 12 weeks, flow-mediated endothelium-dependent dilation, as well as endothelium-independent dilation, was notably increased in the pioglitazone group compared with the placebo. This trend was also present in the pioglitazone group considering total adiponectin, high molecular weight adiponectin, and the ratio of high molecular weight adiponectin /total adiponectin. There was a significant positive correlation between the change in FMD and the change in total adiponectin levels. There was also a positive correlation between the change in endothelium-independent dilation and total adiponectin and high molecular weight adiponectin. The authors have concluded that pioglitazone improved endothelial function in impaired glucose tolerance participants and that the treatment with pioglitazone may reduce the risk of cardiovascular disease in this patient group. The comparable results in regards to the effects of pioglitazone on FMD and adiponectin levels were also obtained in the investigation reported by Tsuchia et al. (2009) [31].

In the multicentre and randomized trial reported by Derosa et al. (2010) [32], it was observed that at full dose, pioglitazone reduced the inflammatory response to a glucose challenge more than acarbose in T2DM patients already treated with maximal doses of sulphonylureas and metformin. The examined population of patients involved 473 Caucasian T2DM subjects (aged ≥18 of either gender) that underwent measurements of different metabolic parameters, as well as markers of ED and vascular inflammation. After a three-month run-in period in which the eligible patients were taking sulphonylureas and metformin at various dosages, the patients were randomized to also receive pioglitazone, titrated up to 45 mg/day (three times a day) or acarbose up to 300 mg/day (three times a day). Assessments were made at the start of titration, after three months (before a first oral glucose tolerance test), after six months, and at the study end (before a second oral glucose tolerance test). Patients were excluded if they had a history of ketoacidosis or had unstable or rapidly progressive diabetic retinopathy, nephropathy, or neuropathy; impaired hepatic function, impaired renal function, or severe anemia. Patients with serious cardiovascular disease or cerebrovascular conditions within six months before the study enrollment were also excluded. The improvement in glucose control was more pronounced with pioglitazone treatment, and this event preceded the improvement in inflammation markers. A decrease in glycated hemoglobin was observed in the pioglitazone group compared to both the baseline value and the acarbose group. The homeostasis model assessment index decreased significantly after the full treatment in the pioglitazone group compared to the end of the titration period and to the acarbose group, whereas IL-6 and TNFa decreased after the full treatment in the pioglitazone group relative to the end of the titration period. In accordance with previous observations, significant high-sensitivity C-reactive protein decrease was obtained after the titration period if compared to the baseline value in the pioglitazone group, and this marker was found to be decreased in the pioglitazone group after the full treatment compared to the end of the titration period and to the acarbose group.

Taking into account that vascular inflammation plays a pivotal role in ED, the effects of pioglitazone and rosiglitazone on the mediators of ED, markers of angiogenesis and inflammatory cytokines in T2DM were investigated by Vijay et al. (2009) [15]. It was concluded that pioglitazone had an advantage over rosiglitazone in lowering lipid and proinflammatory cytokines. A total of 50 subjects matched with predefined inclusion criteria that involved the presence of poorly controlled T2DM (whose HbA_1C_ % exceeded 8.0) with cardiovascular risk factors, age 30–70 years, BMI below 36 kg/m^2^, stable body weight for at least three months before the study, and patients without hepatic or other pre-existing chronic disease. The participants were randomized to receive either pioglitazone, 30 mg or rosiglitazozne, 4 mg in two groups of 20 subjects in each group, still according to the levels of blood sugar, the doses of pioglitazone and rosiglitazone were increased to the maximally effective ones (pioglitazone 45 mg once daily and rosiglitazone 4 mg twice daily). The rest of the 10 participants were randomly selected as controls. The pioglitazone group showed favorable reductions in serum total cholesterol, triglycerides, LDL cholesterol, VLDL cholesterol, and an increase in HDL cholesterol as compared to the rosiglitazone group, after 16 weeks of treatment and also with the control group. The significant reduction in C-reactive protein levels was detected in both treated groups, still more pronounced in the pioglitazone group. The level of serum TNFα was significantly and mildly decreased in the pioglitazone and rosiglitazone groups, respectively. The level of serum markers of angiogenesis, namely VEGF, IL-8, and angiogenin were more increased in the pioglitazone and rosiglitazone group if compared to the control group.

## Final Remarks

Having in mind the serious vascular complications associated with T2DM, an appropriate prevention and treatment of this pathological condition should be a paramount. Accordingly, the widely accepted diagnostic and preventive model of the metabolic response of an individual patient to a meal is the oral glucose tolerance test, which is also a reliable experimental technique to evaluate the modification related to post-prandial glycemia of endothelial stress systemic markers [32]. PPARy activation improves insulin sensitivity, decreases inflammation, plasma levels of free fatty acids, and blood pressure, so indirectly leading to the inhibition of atherogenesis, improvement of ED, and reduction of cardiovascular events [2]. Pioglitazone is an insulin-sensitizing, anti-hyperglycemic agent which stimulates PPARy, yet the vasculoprotective effects seem not to be directly associated to the activation of this nuclear receptor. Nevertheless, positive correlations are currently well documented considering the improvement of different markers related to ED and pioglitazone administration. Still, although pioglitazone has a favorable safety profile, an increased risk for the development/exacerbation of heart failure related to fluid retention [33], as well as a recently reported increase in the incidence of bladder cancer [6] requires a highly individualized approach in the prescription of pioglitazone.

## Abbreviations

ED: Endothelial dysfunction
eNOS: Endothelial nitric oxide synthase
EPC: Endothelial progenitor cells
FMD: Flow-mediated dilation
Hcy: Homocysteine
ICAM-1: Intercellular adhesion molecule-1
IL-6: Interleukin-6
NO: Nitric oxide
PPARy: Peroxisome proliferator-activated receptor-gamma
SHR: Spontaneously hypertensive rats
SNP: Sodium nitroprusside
TNFa: Tumor necrosis factor-a
T2DM: Type 2 diabetes mellitus
VCAM-1: Vascular cell adhesion molecule-1
VEGF: Vascular endothelial growth factor
